# Complex skills are required for new primary health care researchers: a training program responds

**DOI:** 10.1186/s12909-022-03620-3

**Published:** 2022-07-22

**Authors:** Amanda L. Terry, Moira Stewart, Rachelle Ashcroft, Judith Belle Brown, Fred Burge, Jeannie Haggerty, Carol McWilliam, Leslie Meredith, Graham J. Reid, Roanne Thomas, Sabrina T. Wong, and the TUTOR-PHC Program Investigators/Mentors, Robert Van Hoorn

**Affiliations:** 1 Centre for Studies in Family Medicine, Department of Family Medicine, Department of Epidemiology & Biostatistics, Schulich Interfaculty Program in Public Health, Schulich School of Medicine & Dentistry, The University of Western Ontario, Western Centre for Public Health and Family Medicine, 1151 Richmond Street, London, Ontario N6A 3K7 Canada; 2 Centre for Studies in Family Medicine, Department of Family Medicine; Department of Epidemiology and Biostatistics, Schulich School of Medicine and Dentistry, The University of Western Ontario, London, Ontario Canada; 3 Factor-Inwentash Faculty of Social Work, University of Toronto, Toronto, Ontario Canada; 4 Centre for Studies in Family Medicine, Department of Family Medicine, Schulich School of Medicine and Dentistry, The University of Western Ontario, London, Ontario Canada; 5 Department of Family Medicine, Dalhousie University, Halifax, Nova Scotia Canada; 6 Department of Family Medicine, McGill University, Montréal, Québec Canada; 7 Arthur Labatt Family School of Nursing, Faculty of Health Sciences, The University of Western Ontario, London, Ontario Canada; 8 Centre for Studies in Family Medicine, Department of Family Medicine, Department of Psychology, Schulich School of Medicine and Dentistry, The University of Western Ontario, London, Ontario Canada; 9 School of Rehabilitation Sciences, Faculty of Health Sciences, University of Ottawa, Ottawa, Ontario Canada; 10 School of Nursing, Centre for Health Services and Policy Research, University of British Columbia, Vancouver, British Columbia Canada

**Keywords:** Teaching research skills, Education/curriculum development, Interdisciplinary research training, Primary health care research training

## Abstract

**Background:**

Current dimensions of the primary health care research (PHC) context, including the need for contextualized research methods to address complex questions, and the co-creation of knowledge through partnerships with stakeholders – require PHC researchers to have a comprehensive set of skills for engaging effectively in high impact research.

**Main body:**

In 2002 we developed a unique program to respond to these needs - Transdisciplinary Understanding and Training on Research - Primary Health Care (TUTOR-PHC). The program’s goals are to train a cadre of PHC researchers, clinicians, and decision makers in interdisciplinary research to aid them in tackling current and future challenges in PHC and in leading collaborative interdisciplinary research teams. Seven essential educational approaches employed by TUTOR-PHC are described, as well as the principles underlying the curriculum. This program is unique because of its pan-Canadian nature, longevity, and the multiplicity of disciplines represented. Program evaluation results indicate: 1) overall program experiences are very positive; 2) TUTOR-PHC increases trainee interdisciplinary research understanding and activity; and 3) this training assists in developing their interdisciplinary research careers. Taken together, the structure of the program, its content, educational approaches, and principles, represent a complex whole. This complexity parallels that of the PHC research context – a context that requires researchers who are able to respond to multiple challenges.

**Conclusion:**

We present this description of ways to teach and learn the advanced complex skills necessary for successful PHC researchers with a view to supporting the potential uptake of program components in other settings.

## Background

Primary health care (PHC) is repeatedly recognized as a foundational element of health systems, and is often where the majority of health care occurs [1, 2]. Based on international research evidence, we know that countries with a strong PHC care focus have improved population health outcomes [3]. PHC research is important for supporting the provision of high quality PHC and the effective functioning of health care systems [4–6]. Recently, research priorities to address gaps in knowledge have been identified and consensus statements about research in PHC settings (including family medicine and primary care) have been published [4, 6–12]. The knowledge gaps identified speak to the dynamic and evolving nature of the PHC research context, and include a broad spectrum of areas of inquiry, for example, the “basic science” of primary care [7] and the role and impact of health information technology [4, 8]. These gaps also focus on the increased complexity of the care of patients in PHC [13–15] and need for new research methods to address this complexity [16]. To achieve maximum research impact there is a fundamental shift occurring toward the co-creation of knowledge through partnerships among researchers and other stakeholders, including patients and their communities, clinicians, and policy makers [4, 6, 16, 17]. This “knowledge production” approach differs from the more traditional role of academic researchers in knowledge translation [17]. Early career researchers have recently issued a call to action, reflecting on their experiences during the COVID-19 pandemic, articulating the need to focus on the themes of health equity, the modernization of communication strategies to combat mis- and dis-information, and support for innovations such as virtual communities of practice in future research [18].

The perspectives of multiple primary health care disciplines need to be brought to bear to address these gaps. This includes researchers working in interdisciplinary teams, and with multiple stakeholders [9, 16, 19, 20]. Therefore, PHC researchers need to have a complex and comprehensive set of skills for engaging effectively in high impact research. These individuals need to be able to span the boundaries of the academic world and those of the broader health care system, and be prepared to work in interdisciplinary teams in non-academic settings [21–23]. Despite this, training of PHC researchers still occurs in the silos of their own discipline, with few opportunities for individuals from different PHC disciplines to come together.

A unique, well-established training program for research in PHC exists which builds capacity to address these needs - Transdisciplinary Understanding and Training on Research – Primary Health Care (TUTOR-PHC) [24, 25]. The program’s goals are to train a cadre of PHC researchers, clinicians, and decision makers in interdisciplinary research to aid them in tackling current and future challenges in PHC and lead collaborative interdisciplinary research teams. The program targets three groups: 1) early career researchers (e.g. PhD trainees and Post-Doctoral Fellows) from different PHC disciplines; 2) mid-career clinicians; and 3) decision makers at various career stages. The program is unique because its trainees and mentors come from across Canada and from a multiplicity of disciplines [26].

Having reached a milestone of 189 graduates (23 of whom are international trainees and 166 Canadian), and 15 years of delivering the TUTOR-PHC program, in this paper we describe ways to teach and learn the advanced complex skills necessary for successful PHC researchers, with a view to supporting the potential uptake of program components in other settings. This paper therefore provides an overview of TUTOR-PHC, describes the concepts and principles underlying the program, includes examples of curriculum components related to these concepts and principles, and presents a summary of the program evaluation findings. Finally, we discuss the need for such programs.

### Program structure and overview

The focus of the TUTOR-PHC program is on learning how to conduct and meaningfully apply interdisciplinary research that engages patients, communities, practitioners and policy-makers. TUTOR-PHC brings together trainees and mentors from different PHC-oriented disciplines in a year-long training program. Fourteen trainees participate each year; these trainees are divided into two equal groups for the purposes of the program. Program mentors include a group of academic faculty members, policy-makers, and patient partners. Mentors develop and deliver the curriculum content, which includes an in-person Symposium held over 4 days, followed by on-line activities that include workshops about research topics, peer feedback on the trainee’s research project, and the trainees working as an interdisciplinary team to develop a mock-grant proposal. The total time is approximately equivalent to a year-long (2 semester) graduate course.

### Development and implementation of educational approaches and curriculum components

In developing the TUTOR-PHC curriculum, we wrestled with the necessary content (the “what” of research) such as mixed-methods designs and the measurement of outcomes. It became clear that the process (the “how” of research) such as working with different disciplines and engaging with patients and policy-makers was equally important. Parallel to this insight was the realization that the teaching and learning had to be experiential in order for the trainees to fully integrate the skills they were learning. Therefore, we apply a set of seven educational approaches guided by evidence from the literature: developing research skills [27]; acquiring explicit knowledge [28]; absorbing tacit knowledge [28, 29]; co-creating learning collaboratively [28]; integrating knowledge through critical reflection [30, 31]; creating a community of scholars; and educating for capability [32]. The intersection of these approaches with the seven curriculum components of the TUTOR-PHC program is illustrated in Table 1.

**Table 1:** Seven curriculum components and their educational approaches

	Education Content- the ‘What’			Education Processes – the ‘How’			
Curriculum Components	Research Skills Development^a^	Explicit Knowledge^b^	Tacit Knowledge^c^	Collaborative Co-created Learning^d^	Critical Reflection^e^	Community of Scholars^f^	Educate for Capability^g^
1) What is Primary Health Care	✓	✓	✓	✓	✓		
2) Research Methodologies	✓	✓	✓	✓			✓
3) Knowledge Translation	✓	✓	✓	✓	✓		✓
4a) Interdisciplinary Collaborative Team Development Workshop	✓	✓	✓				
4b) Interdisciplinary Collaborative Team Development Discussion	✓		✓	✓	✓	✓	✓
5a) Interdisciplinary Grant Proposal Writing Workshop		✓					
5b) Interdisciplinary Grant Proposal Writing Discussion	✓		✓	✓	✓	✓	✓
6. Policy-Maker Engagement		✓	✓	✓	✓	✓	✓
7. Patient Engagement		✓	✓	✓	✓	✓	✓

✓ means the Component is addressed using the particular approach

^a^ Research Skill Development framework is an aid for educators developing a curriculum to take students from low levels of student autonomy (Level 1 = closed inquiry and high degree of structure) to high levels (Level V = open inquiry with self-determined guidelines) [27]. TUTOR-PHC provides opportunities culminating in Component 4b & 5b which provide Level V training

^b^ Explicit knowledge is: “formal (mathematical equations, scientific papers and train timetables); can be expressed in symbols (codified); and is therefore easy to communicate, transfer and measure” [28]. See also Nutley et al. [29]

^c^ Tacit Knowledge is “informal (as in ‘knowing the ropes’) and is difficult to codify and transfer between individuals”. It has three inherent properties: “inextricably woven with … experiences and situational contexts; dependent for its meaning on interpretation… by individuals in a particular context; the person… needs to have some prior knowledge and experience… for the new knowledge to make sense” [28]

^d^ Collaborative Co-creative Learning is a “social process involving the active construction of new knowledge and understandings through group interaction and peer discussion” [28]

^e^ Critical reflection, advocated by Schon [30] and Mezirow [31] for “workplace learning” on “ill-defined and messy problems” in the real world, permits an opening of “meaning perspectives” allowing more integrative learning i.e., learning to put the new knowledge into the practice of research [33]. See also McWilliam [34]; Taylor & Hamdy [35]

^f^ Community of Scholars means that TUTOR-PHC is explicit in its goal to create a community of PHC researchers which will be an ongoing resource for all trainee graduates. Components 4b and 5b enhance the community-building

^g^ Educate for Capability means to provide appropriate learning for complex contexts, i.e., “for its applicability to problems in the work environment… (in the form of) transferable problem-solving strategies” [28, 32]

For developing Research Skills, the curriculum moves trainees from low levels of autonomy (Level 1 = closed inquiry and high degree of structure) through to high levels (Level V = open inquiry with self-determined guidelines) [27]. Lectures provide a high degree of structure while one-on-one or group discussions encourage open inquiry and self-directed experiences.

Explicit Knowledge is: “formal (mathematical equations, scientific papers…); can be expressed in symbols (codified); and is therefore easy to communicate, transfer and measure” [28]. Acquiring explicit knowledge occurs through structured learning from interactive lectures on: the history of definitions of PHC; mixed methods; and interdisciplinary concepts in the literature.

Tacit Knowledge is defined as “informal (as in ‘knowing the ropes’) and is difficult to codify and transfer between individuals” [28]. Tacit Knowledge has three elements: “[a] inextricably woven with… experiences and situational contexts; [b] dependent for its meaning on interpretation… by individuals in a particular context; [and c] the person… needs to have some prior knowledge and experience… for the new knowledge to make sense” [28, 29]. Imparting tacit knowledge is a key challenge for educators. Recognizing and absorbing it is also a challenge for trainees. TUTOR-PHC provides opportunities for mentors and trainees to share their experiences in an atmosphere of conversational exchange, which appear to be informal but are actually highly scripted with mentors prepared to offer key messages. For example, through exercises to expose trainees to real-life situations in context during either role-playing or presenting their own experiences, they learn to present and listen to others’ experiences. These exercises which enhance tacit learning are key.

Co-creative Learning Collaboratively is a “social process involving the active construction of new knowledge and understandings through group interaction and peer discussion” [28]. Mentors facilitate constructive group process so that each small discussion group of trainees can co-create an interdisciplinary research proposal. This difficult work is guided by a goal-oriented framework in which mentors keep in mind expectations for each step, observe, and reflect back to trainees, how the group is navigating the process.

Critical Reflection, advocated by Schon [30] and Mezirow [31] for “workplace learning” on “ill-defined and messy problems” in the real world, permits an opening of “meaning perspectives” allowing more integrative learning; that is learning to put the new knowledge into the practice of research [33, 34] Facilitating critical reflection occurs during group discussions and written exercises completed independently by each trainee; the goal is to integrate the learnings and create a readiness in the trainee for real-world experiences.

We set an explicit goal to create a Community of Scholars (early researchers) which will be an ongoing resource for all trainee graduates. The development of this community is fostered through an exercise where each trainee describes their disciplinary perspective and what their discipline brings to the research process. Thus, the trainee group understands the scholarly role of each member, setting the stage for future collaborative activities. Creating a community of scholars also requires an ongoing commitment of time and resources for two purposes: ongoing mentoring over years; and, follow-up alumnae gatherings and symposia both formal and informal.

Educating for Capability refers to handling “problems in the work environment… (using) transferable problem-solving strategies” [28, 32]. The task of the mentors is to imagine and describe real-world contexts and give the trainees enough information about the roles of the various players for them to assume a role and practice it. Work in developing scenarios requires vast experience on the part of the mentors, taken from their own research careers and necessitates as much work as developing a traditional curriculum. The principles of developing these scenarios will be presented next.

The principles for the curriculum are: include a real-world context; be task oriented; provide opportunity for the trainee both to reveal his or her own experience (self-knowledge) and to listen to experiences of others; encourage a collaborative process leading to co-creation of knowledge [36, 37]; allow for tacit exchanges; and foster self-reflection.

### Examples of TUTOR-PHC curriculum components that exemplify the curricular principles and educational approaches

#### Example 1: interdisciplinary research teams

This key part of the TUTOR-PHC curriculum is multi-faceted and illustrates many of the principles above. This example maps to curriculum components 4a, 4b and 5a, 5b illustrated in Table 1. A didactic lecture on the concepts of interdisciplinary care and research from the literature begins the process. Next, each mentor describes his or her own discipline (e.g. nursing, psychology, family medicine) with a small group of trainees who are provided with three questions to ask mentors: one about their role on an interdisciplinary research team; second about the unique features their discipline brings to the interdisciplinary research; and third about their definition of their discipline and its relevance to PHC research.﻿ This exercise accentuates real-world diversity within and between disciplines. In this part of the exercise, trainees listen and learn about their own discipline and other disciplines.

In the next part of the exercise, trainees come together in their small discussion groups and are asked to define and take on the mantle of their discipline, while at the same time noting similarities and differences across disciplines. The trainees listen and practice responding to their peers’ descriptions with comments from their own discipline’s perspective. This exercise is difficult perhaps because the trainees have been trained in single discipline graduate programs such as epidemiology or psychology and have rarely been exposed to teamwork with different disciplines. Another exercise that requires a great deal of preparation is the task of an interdisciplinary group of trainees co-writing a grant application together, guided by two mentors. This process takes 4 h of face-to-face time during the Symposium and seven to nine weeks of on-line work. Each trainee takes responsibility for leading the writing and on-line discussion for 1 week as the proposal is being developed. Two essential structures are: 1) the fictional call for proposals; and 2) the table of contents of the sections of the grant proposal that must be written on-line, each section led by one trainee and given a week to complete. Facilitation of the group discussion process is rigorously guided using the mentors’ tacit knowledge and examples.

This complex series of sessions on interdisciplinary team development illustrates how to educate for capability through strong facilitation using real-world exercises and rigorous structures such as a grant proposal call and a facilitation guide for mentors.

#### Example 2: patient/community engagement & policy-maker engagement

Preparing early-career PHC researchers to develop research relationships with multiple stakeholder groups is important. As the TUTOR curriculum evolved, a component on engaging with policy-makers was added in 2010. Another addition (initiated in 2017) focuses on engaging with patients/community. Examples of these additions are described below; they map to curriculum components 6 and 7 listed in Table 1.

The policy-maker session is divided into two components. First is a question-and-answer panel session between researchers and policy-makers (e.g. Director of a Regional Health Authority) designed to allow the trainees to understand a policy-maker’s world, e.g. their motivations, the demands upon them, how they interact with researchers. This interaction builds trainees’ capacity to effectively engage and develop relationships with the policy-makers. The second half of the session involves the trainees each developing and practicing delivering a 45-second “elevator pitch” about their research project for a policy-maker.

The patient/community engagement session (co-created and presented with TUTOR’s patient partners) is divided into three components. First are didactic presentations describing approaches to patient and community engagement in research. Second, mentors present to trainees, posters of their own work illustrating patient and community engagement in research. Trainees learn how this type of research can occur in practice, thus gaining explicit knowledge. Mentors share their tacit knowledge by discussing their experiences of addressing real-world research challenges – “the story behind the poster”. Third is a facilitated small group discussion and an exercise where the trainees interact with patients/community members who are engaged in research.

Many of the trainees will have had little to no experience with patient and community engagement in research. The aforementioned exercise prompts the trainees to actively consider how they could integrate patient/community engagement into their own work. Small group discussion ensues during which trainees and the patient/community member leaders share experiences. Trainees often raise potential challenges, and patient/community members offer solutions based on their knowledge and expertise.

This sequence of sessions, held at the end of the on-site component of the curriculum enable trainees to integrate their learnings and to apply them in their own context [32].

##### TUTOR-PHC program evaluation summary

TUTOR-PHC has a multi-faceted approach to evaluating the program’s impact, including assessments of trainee experiences and interdisciplinary research activities, and research productivity over time. Since these are program evaluation activities, they are considered exempt from human ethics review in accordance with Article 2.5 of the Tri-Council Policy Statement: Ethical Conduct for Research Involving Humans [38].

Taken together, evaluation results indicate that TUTOR-PHC is achieving its goals. The results of an evaluation questionnaire administered at the end of the program year demonstrate that trainees’ overall program experiences are very positive (mean 3.51, scale 1- poor to 4 -excellent). The vast majority (95%) believed that the TUTOR-PHC program provided appropriate benefit for the time the trainee devoted to the program. In response to yes/no questions regarding the role of TUTOR-PHC in increasing trainee understanding of interdisciplinary research, all respondents indicated yes (100%); 88% responded in the affirmative that the program had increased their interdisciplinary research activity. The vast majority of trainees viewed the TUTOR-PHC program as assisting in the development of their careers with respect to interdisciplinary research to date (81%) and in the future (100%). Despite the questionnaire being administered at an early stage (at the end of the program year) almost a quarter of trainees (23%) had forged new collaborations with researchers from different disciplinary backgrounds, and a more than a third (35%) had developed collaborations with other TUTOR-PHC trainees, supervisors, or mentors. Over time, these relationships continue to grow, resulting in joint authorship on publications, collaborations on successful grant proposals, and trainees becoming mentors in TUTOR-PHC [24–26]. TUTOR-PHC trainees are highly productive researchers and leaders in PHC [24–26, 39] and have described the impact of the program from their own perspectives [40, 41].

##### Reflections on the TUTOR-PHC program and lessons learned

The current PHC research context requires that researchers have a complex set of skills and competencies preparing them to engage effectively in research. There is both an emphasis on interdisciplinary research and a recognition of its growing importance [23, 42–44], yet there is more to do to support interdisciplinary research capacity building [23, 43, 45, 46]. Competencies such as interdisciplinary work, collaboration, and networking are important for health services researchers, [23] yet graduate education is usually focused on developing an individual’s expertise and professional identity within a particular discipline. There are few opportunities for PHC researchers to come together to develop the skills and competencies needed to conduct interdisciplinary research and to engage stakeholders. This poses a problem for early-career researchers who want to engage, and for their supervisors/mentors who would like to prepare them for this work.

TUTOR-PHC has responded to this challenge by developing and implementing a training program which is designed to help trainees learn the complex skills necessary to be successful interdisciplinary PHC researchers. While all of the components of TUTOR-PHC are important, we focused on two examples - interdisciplinary teams and engagement with patients, communities, and policy makers - to highlight how the educational approaches and principles are operationalized in the program. It is the synergistic nature of all the approaches coming together in a multi-faceted whole, underpinned by the program principles that allow the full impact of TUTOR-PHC to be realized.

TUTOR-PHC is structured such that there are multiple methods of content delivery (e.g. didactic, small group, experiential), multiple platforms for learning (e.g. in-person, on-line), exposure to multiple teachers/mentors, and multiple areas of learning (e.g. interdisciplinary teamwork, research methodology). Taken together, the structure of the program, content, educational approaches, and principles, represent a complex whole. This complexity parallels that of the PHC research context – a context that requires researchers who are able to respond to its challenges.

There are two lessons learned during the 15 years of the program that requiring emphasis. First, the TUTOR-PHC program demonstrates how to educate for collaborative skills and success. The program gives trainees skills that they would not have encountered otherwise in their training, allowing them to become effective, high impact interdisciplinary PHC researchers. Second, importance is placed on the program being able to respond to emerging needs in the PHC research context. Periodically examining this context has inspired TUTOR-PHC mentors to revise the curriculum to include components such as engaging new partners in research; representing an evolution of the program.

## Conclusion

The experience of TUTOR-PHC sets trainees on a path to success in their interdisciplinary research. The program has responded to the evolving nature of the PHC context and research training needs. Interdisciplinary research yields answers to questions in PHC that we would not otherwise find; TUTOR-PHC fulfills the need to train researchers to work in this milieu. We present this description of ways to teach and learn the advanced complex skills necessary for successful PHC researchers with a view to supporting the potential uptake of program components in other settings.

## Data Availability

The datsets generated and/or analyzed are not publicly available because they contain information that could compromise trainee privacy but are available from the corresponding author on reasonable request.

## Abbreviations

PHC: Primary health care
TUTOR-PHC: Transdisciplinary Understanding and Training on Research - Primary Health Care

## References

[1] Stewart M, Ryan B (2015). Ecology of health care in Canada. Can Fam Physician.

[2] Green LA, Fryer GE, Yawn BP, Lanier D, Dovey SM (2001). The ecology of medical care revisited. N Engl J Med.

[3] Starfield B, Shi L, Macinko J (2005). Contribution of primary care to health systems and health. Milbank Q.

[4] Bierman AS, Tong ST, McNellis RJ (2022). Realizing the dream: the future of primary care research. Ann Fam Med.

[5] Hobbs R (2019). Is primary care research important and relevant to GPs?. Br J Gen Pract.

[6] Kluge H, Kelley E, Theodorakis PN, Barkley S, Valderas JM (2018). Forty years on from Alma Ata: present and future of primary health care research. Prim Health Care Res Dev.

[7] Westfall JM, Wittenberg HR, Liaw W (2021). Time to invest in primary care research—commentary on findings from an independent congressionally mandated study. J Gen Intern Med.

[8] O’Neill B, Aversa V, Rouleau K, Lazare K, Sullivan F, Persaud N. Identifying top 10 primary care research priorities from international stakeholders using a modified Delphi method. PLoS One. 2018;13(10):e0206096. 10.1371/journal.pone.0206096 PMC6201922 30359391

[9] Montesanti S, Robinson-Vollman A, Green LA. Designing a framework for primary health care research in Canada: a scoping literature review. BMC Fam Pract. 2018;19(1):144. 10.1186/s12875-018-0839-x PMC6116436 30157764

[10] Hirschhorn LR, Langlois EV, Bitton A, Ghaffar A. What kind of evidence do we need to strengthen primary healthcare in the 21st century? BMJ Glob Health. 2019;4(Suppl 8):e001668. 10.1136/bmjgh-2019-001668 PMC6703285 31478031

[11] Burgers JS, Wittenberg J, Keuken DG, Dekker F, Hohmann FP, Leereveld D (2019). Development of a research agenda for general practice based on knowledge gaps identified in Dutch guidelines and input from 48 stakeholders. Eur J Gen Pract.

[12] Palagyi A, Dodd R, Jan S, Nambiar D, Joshi R, Tian M, et al. Organisation of primary health care in the Asia-Pacific region: developing a prioritised research agenda. BMJ Glob Health. 2019;4(Suppl 8):e001467. 10.1136/bmjgh-2019-001467 PMC6703300 31478022

[13] Salisbury C, Lay-Flurrie S, Bankhead CR, Fuller A, Murphy M, Caddick B, et al. Measuring the complexity of general practice consultations: a Delphi and cross-sectional study in English primary care. Br J Gen Pract. 2021;71(707):e423–31. 10.3399/BJGP.2020.0486 PMC8049201 33824162

[14] Webster F, Rice K, Bhattacharyya O, Katz J, Oosenbrug E, Upshur R (2019). The mismeasurement of complexity: provider narratives of patients with complex needs in primary care settings. Int J Equity Health.

[15] Upshur REG, Tracy S (2008). Chronicity and complexity. Can Fam Physician.

[16] Bayliss EA, Bonds DE, Boyd CM, Davis MM, Finke B, Fox MH (2014). Understanding the context of health for persons with multiple chronic conditions: moving from what is the matter to what matters. Ann Fam Med.

[17] Greenhalgh T, Jackson C, Shaw S, Janamian T (2016). Achieving research impact through co-creation in community-based health services: literature review and case study. Milbank Q.

[18] Chisholm A, Wang J, Bonnell LN, Duwe E, Gilfoyle M, Kueper JK (2022). From NAPCRG: primary care research through the lens of NAPCRG's trainee committee: a year of reflection in a pandemic and a call to action. Ann Fam Med.

[19] Ponka D, Coffman M, Fraser-Barclay KE, Fortier RDW, Howe A, Kidd M, et al. Fostering global primary care research: a capacity-building approach. BMJ. Glob Health. 2020;5(7):e002470. 10.1136/bmjgh-2020-002470 PMC7337619 32624501

[20] Cheraghi-Sohi S, Perry M, Wallace E, Wallis KA, Geraghty AW, Joling KJ (2018). A future in primary care research: a view from the middle. Br J Gen Pract.

[21] McMahon M, Habib B, Tamblyn R (2019). The career outcomes of health services and policy research doctoral graduates. Healthc. Policy..

[22] Bornstein S, Heritage M, Chudak A, Tamblyn R, McMahon M, Brown AD (2018). Development of enriched core competencies for health services and policy research. Health Serv Res.

[23] Sibbald SL, Peirson L, Boyko J (2015). Squaring circles: the gap for interdisciplinary trainees in a discipline-driven academy. IJHE..

[24] Stewart M, Wuite S, Ramsden V, Burge F, Beaulieu M-D, Fortin M (2014). Transdisciplinary understandings and training on research: successfully building research capacity in primary health care. Can Fam Physician.

[25] Stewart M, Reid G, Brown JB, Burge F, Dicenso A, Watt S (2010). Development and implementation of training for interdisciplinary research in primary health care. Acad Med.

[26] Terry AL, Brown JB, Van Hoorn R, Stewart M (2018). TUTOR-PHC program co-investigators. Evolution and 15-year effect of a pan-Canadian training program. Can Fam Physician.

[27] Willison J, O’Regan K (2007). Commonly known, commonly not known, totally unknown: a framework for students becoming researchers. High Educ Res Dev.

[28] Greenhalgh T, Russell J (2006). Promoting the skills of knowledge translation in an online master of science course in primary health care. J Contin Educ Heal Prof.

[29] Nutley S, Walter I, Davies HTO (2003). From knowing to doing: a framework for understanding the evidence-into-practice agenda. Evaluation..

[30] Schon D (1983). The reflective practitioner.

[31] Mezirow J (1996). Contemporary paradigms of learning. Adult Educ Q.

[32] Fraser SW, Greenhalgh T (2001). Complexity science: coping with complexity: educating for capability. BMJ..

[33] Fenwick TJ (2000). Expanding conceptions of experiential learning: a review of the five contemporary perspectives on cognition. Adult Educ Q.

[34] McWilliam CL (2007). Continuing education at the cutting edge: promoting transformative knowledge translation. J Contin Educ Heal Prof.

[35] Taylor DCM, Hamdy H (2013). Adult learning theories: implications for learning and teaching in medical education: AMEE guide no. 83. Med Teach.

[36] Stewart M, Brown JB, Freeman TR, McWilliam CL, Mitchell J, Brown L, Stewart M, Brown JB, Weston WW, McWhinney IR, Freeman TR (2014). Part four: the health care context and patient-centered care. Team-centered approach: how to build and sustain a team. Patient-Centred medicine transforming the clinical method.

[37] D’Amour D, Goulet L, Labadie J-F, Martín-Rodriguez LS, Pineault R (2008). A model and typology of collaboration between professionals in healthcare organizations. BMC Health Serv Res.

[38] Canadian Institutes of Health Research, Natural Sciences and Engineering Research Council of Canada, Social Sciences and Humanities Research Council of Canada (2018). Tri-Council policy statement: ethical conduct for research involving humans.

[39] Transdisciplinary Understanding and Training on Research- Primary Health Care. Available from: https://www.uwo.ca/fammed/csfm/tutor-phc/index.html. Accessed 7 Mar 2022.

[40] Perreault K, Boivin A, Pauzé E, Terry AL, Newton C, Dawkins S (2009). Interdisciplinary primary health care research training through TUTOR-PHC: the insiders’ view. J Interprof Care.

[41] Contant E, Nicholson K. Transdisciplinary collaboration: the future of primary health care. Health Sci Inq. 2019;5(1) Available from: https://www.healthscienceinquiry.com/index.php/hsi/article/view/172. Accessed 18 Apr 2022.

[42] Clarke D, Hawkins R, Sadler E, Harding G, Forster A, McKevitt C (2012). Interdisciplinary health research: perspectives from a process evaluation research team. Qual Prim Care.

[43] Gill SV, Vessali M, Pratt JA, Watts S, Pratt JS, Raghavan P (2015). The importance of interdisciplinary research training and community dissemination: interdisciplinary research education. Clin Transl Sci.

[44] Hall JG, Bainbridge L, Buchan A, Cribb A, Drummond J, Gyles C (2006). A meeting of minds: interdisciplinary research in the health sciences in Canada. CMAJ..

[45] Nicholson K, Ganann R, Bookey-Bassett S, Garland Baird L, Garnett A, Marshall Z (2020). Capacity building and mentorship among pan-Canadian early career researchers in community-based primary health care. Prim Health Care Res Dev.

[46] Russell G, Goodyear-Smith F, Mash B (2019). A systems approach to building research capacity: individuals, networks and culture. How to do primary care research.

